# Development and Validation of a Predictive Model for Toxicity of Neoadjuvant Chemoradiotherapy in Rectal Cancer in the CAO/ARO/AIO-04 Phase III Trial

**DOI:** 10.3390/cancers14184425

**Published:** 2022-09-12

**Authors:** Markus Diefenhardt, Daniel Martin, Ethan B. Ludmir, Maximilian Fleischmann, Ralf-Dieter Hofheinz, Michael Ghadimi, Rebekka Kosmala, Bülent Polat, Tim Friede, Bruce D. Minsky, Claus Rödel, Emmanouil Fokas

**Affiliations:** 1 Department of Radiotherapy and Oncology, University of Frankfurt, 60596 Frankfurt, Germany; 2 Frankfurt Cancer Institute, 60596 Frankfurt, Germany; 3 German Cancer Research Center (DKFZ), German Cancer Consortium (DKTK), Partner Site, Frankfurt, 69120 Heidelberg, Germany; 4 Division of Radiation Oncology, The University of Texas MD Anderson Cancer Center, Houston, TX 77054, USA; 5 Department of Medical Oncology, University Hospital Mannheim, 68167 Mannheim, Germany; 6 Department of General and Visceral Surgery, University Medical Center Göttingen, 37075 Göttingen, Germany; 7 Department of Radiation Oncology, University Hospital Würzburg, 97080 Würzburg, Germany; 8 Department of Medical Statistics, University Medical Center Göttingen, 37075 Göttingen, Germany

**Keywords:** rectal cancer, toxicity, neoadjuvant, chemoradiotherapy, risk score

## Abstract

**Simple Summary:**

Intensified neoadjuvant treatment in rectal cancer can enhance tumor regression and improve survival. However, treatment-related side effects can compromise the success of these treatments by leading to premature discontinuation of therapy. We developed and validated a predictive model for the occurrence of high-grade treatment-related toxicity based on 1236 patients treated within the CAO/ARO/AIO-04 randomized phase III trial. Our prediction score, based on gender, BMI, and emotional function significantly correlated with the occurrence of higher-grade toxicity. Our model could help to identify vulnerable patients at risk for treatment-related high-grade toxicity and provide them with additional supportive treatment options early to improve treatment compliance and oncological outcome

**Abstract:**

Background: There is a lack of predictive models to identify patients at risk of high neoadjuvant chemoradiotherapy (CRT)-related acute toxicity in rectal cancer. Patient and Methods: The CAO/ARO/AIO-04 trial was divided into a development (n = 831) and a validation (n = 405) cohort. Using a best subset selection approach, predictive models for grade 3–4 acute toxicity were calculated including clinicopathologic characteristics, pretreatment blood parameters, and baseline results of quality-of-life questionnaires and evaluated using the area under the ROC curve. The final model was internally and externally validated. Results: In the development cohort, 155 patients developed grade 3–4 toxicities due to CRT. In the final evaluation, 15 parameters were included in the logistic regression models using best-subset selection. BMI, gender, and emotional functioning remained significant for predicting toxicity, with a discrimination ability adjusted for overfitting of AUC 0.687. The odds of experiencing high-grade toxicity were 3.8 times higher in the intermediate and 6.4 times higher in the high-risk group (*p* < 0.001). Rates of toxicity (*p* = 0.001) and low treatment adherence (*p* = 0.007) remained significantly different in the validation cohort, whereas discrimination ability was not significantly worse (DeLong test 0.09). Conclusion: We developed and validated a predictive model for toxicity using gender, BMI, and emotional functioning. Such a model could help identify patients at risk for treatment-related high-grade toxicity to assist in treatment guidance and patient participation in shared decision making.

## 1. Introduction

Patients diagnosed with locally advanced rectal cancer are typically treated with an intensive and lengthy multimodal treatment approach, including neoadjuvant long-course chemoradiotherapy (CRT) (or short-course radiotherapy), followed by total mesorectal excision surgery and optional adjuvant chemotherapy [1,2,3].

Additional sequential neoadjuvant chemotherapy (CT) has been added as part of the total neoadjuvant treatment (TNT) approach in several trials that demonstrated enhanced local tumor regression and improved disease-free survival, mainly by decreasing the risk of distant metastases [4,5,6,7]. Further, a clinical complete remission after CRT/TNT may offer the possibility of a watch-and-wait approach for organ preservation, avoiding surgical morbidity [8,9,10]. However, intensified treatment is often associated with high-grade organ or hematologic toxicity that can impair treatment adherence and consequently, negatively impact long-term oncologic outcomes [11,12,13]**,** as well as quality of life (QoL) [14,15,16].

Although a higher incidence of acute toxicity to CRT has been reported in female [17,18] and underweight patients [19,20] with rectal cancer, no prediction models based on pretreatment parameters, including baseline results of quality-of-life questionnaires, have yet been reported. Such a model may help identify patients at highest risk for treatment-related high-grade toxicity to assist treatment guidance and patient participation in shared decision making [21].

In the present analysis, we aimed to develop and validate a prediction model for high-grade CRT-related toxicity. The model is based on post hoc analysis of a large cohort of 1236 patients with rectal cancer treated in the CAO/ARO/AIO-04 phase III randomized trial. In this trial, the addition of oxaliplatin to 5-FU-based preoperative CRT resulted in a significant improvement in the primary endpoint, DFS, compared to the standard arm [22]. We have defined three quality characteristics that our prediction model should comply with: (1) the prediction model should be applicable to at least two-thirds of the trial cohort, (2) the defined risk groups should be significantly correlated with the occurrence of high-grade toxicity, and (3) the discrimination ability in the validation cohort should not be significantly weaker compared to the development cohort.

## 2. Materials and Methods

The CAO/ARO/AIO-04 trial (ClinicalTrials.gov, number NCT00349076) was a multicenter, open-label, randomized phase III trial that recruited 1265 patients between July 2006 and February 2010. The design, treatment plan, and clinical outcomes have been previously published [22]. For the present post hoc secondary analysis, the study cohort was arbitrarily divided into a development and a validation cohort, where patients treated in Bavaria were included in the validation cohort (n = 405), and patients treated in the other German states were included in the development cohort (n = 831). Neoadjuvant CRT-related toxicity was defined according to CTCAE v3.0. Analyzed CTCAE terms are listed in Table S1. Patient classification was based on the highest reported toxicity grade: patients without grade 3 or 4 adverse events were assigned to the low toxicity group, and patients with at least one grade 3 or 4 adverse event were assigned to the high toxicity group.

Calculation of multiple-item or single-measure scores based on the QLQ-C30 and QLQ-C38 German-translated pre-treatment QoL questionnaires followed EORTC recommendations and were linearly transformed to a 0–100 scale [23]. For multiple items, two scores were calculated: one that only included patients who answered all necessary questions, and one including patients who answered at least 50% of the questions. A higher functional score (e.g., physical functioning, role functioning, etc.) indicates a higher level of function. For the symptom scale (e.g., fatigue, pain, etc.), a higher score represents a higher level of symptoms. Regarding the global health status (GHS), a higher score represents a higher QoL [23,24].

In the development cohort, the association between clinicopathologic characteristics, pretreatment blood parameters, baseline QoL questionnaire scores, and toxicity was examined using the chi-square test or the Mann–Whitney U test for continuous variables. The methodology for the best subset selection approach, as well as the development and validation of the predictive model, were based on those previously reported in the work of [25,26,27]**,** and are described in detail in Supplementary Methods.

Analyses were performed using SPSS^®^27 and the R-Project for statistical computing using the following packages/functions: bestglm, pROC, survminer, ggplot2, and functions: aucadj and modelvalid of the GmAMisc package. All statistical tests were two-sided, and *p*  <  0.05 was considered statistically significant.

## 3. Results

### 3.1. Development and Validation Cohort

Of the 1236 patients, 831 (treated in states other than Bavaria) and 405 (treated in Bavaria) were arbitrarily assigned to the development and validation cohorts, respectively. Among the 831 patients in the development cohort, 155 (18.7%) experienced high-grade toxicity during CRT. Diarrhea (n = 67), pain (n = 23), infection (n = 17), nausea/vomiting (n = 17), mucositis (n = 15), radiation dermatitis (n = 14), leukopenia (n = 12), and proctitis (n = 10) were the most common grade 3–4 toxicities (Table S1). Female patients experienced high-grade toxicity more often (*p* < 0.001), and a higher BMI was associated with a lower risk of toxicity (*p* = 0.001, Table 1). Lower pretreatment levels of erythrocytes (*p* = 0.003) and reduced GFR (*p* = 0.017) were also associated with high-grade toxicity. Besides GFR (AUC 0.424) and levels of erythrocytes (AUC 0.438), alkaline phosphatase (AUC 0.545), bilirubin (AUC 0.459), Hb (AUC 0.457), and neutrophils (*p* = 0.542) also reached a potentially meaningful discrimination level (Table S2).

### 3.2. Association between QLQ and Toxicity

Initially, the association between baseline answers to single questions of QLQ-CR30 and QLQ-CR38 and toxicity were analyzed, as listed in Table S3. Answers to LQ30 [GHS: “How would you rate your overall quality of life during the past week?”] (AUC 0.398), and LQ22 [Emotional functioning: “Did you worry?”] (AUC 0.617), showed the best discrimination ability, whereas answers to LQ52 [Female sexual problems: ”Did you have a dry vagina during sexual intercourse?”] (AUC 0.640), and LQ62 [Stoma Bag: “Were you afraid that other people might hear your stoma?”] (AUC 0.634), showed superior discrimination abilities, but these latter questions were only answered by 70 women and 129 patients who required a stoma prior to CRT, respectively (Table S3).

In the next step, the association between calculated and linear transformed function or symptoms scores and toxicity were investigated. The average score for baseline emotional function, social function, and body image (including patients with missing items) was significantly lower for patients that experienced high-grade toxicity (*p* < 0.001, *p* = 0.007, *p* = 0.029, respectively). Baseline symptoms scores were significantly higher in patients with fatigue (*p* = 0.007), pain (*p* = 0.030), insomnia (*p* = 0.035), and appetite loss (*p* = 0.049). Patients without high-grade organ toxicity had a significantly better baseline GHS (*p* = 0.002). The best discrimination ability was achieved by emotional function scores (AUC 0.352), global health status (AUC 0.410), and female sexual problems scores (AUC 0.625), but only 70 women answered these questions (Table S4). The calculation of the functional and symptoms scores, including patients who answered at least 50% of the necessary questions, did not significantly change the association between baseline QoL scores and toxicity. Hence, we decided to use these in the regression modeling following the EORTC recommendation for adjusted calculation [23].

### 3.3. Binary Logistic Regression Models for Clinical Characteristics, Blood Parameters, and QoL

Binary logistic regression models only including clinicopathologic characteristic, pretreatment blood parameter, or baseline QoL questions were initially analyzed independently. Best subset selection for clinicopathologic characteristics identified a model including treatment arm, gender, ECOG, BMI, cN-category, grading, and localization, which achieved an AUC of 0.629. Only two parameters, gender and BMI, were significant in this model. A binary regression model including only these two parameters achieved an AUC of 0.619. Of the 15 different pretreatment blood parameters included in a best-subset selection model, only GFR was significant (*p* < 0.043), whereas erythrocytes, urea, and neutrophils had a *p*-value < 0.1.

In a second model, created by physician decision (that included erythrocytes, urea, GFR and neutrophils), only GFR and neutrophils had a *p* value < 0.1. From 68 baseline QoL-related questions, 15 questions with a potential association to toxicity and of clinical relevance were included in a best-subset selection analysis. The best-subset selection model included 9 questions, but only LQ22 [Emotional functioning: “Did you worry?”] and LQ30 [GHS: “How would you rate your overall quality of life during the past week?”] had a *p*-value < 0.1, and the complete model achieved an AUC of 0.655. Following testing of the single questions, the function and symptoms scores were assessed. Emotional functioning and body image were the only variables with a *p* value < 0.1 in the proposed model.

Based on these results, a third model addressing QoL was tested, including physical, emotional, cognitive and social functioning, body image, future perspectives, fatigue, pain, appetite loss, and global health status, which achieved an AUC of 0.623. In all described models, correlations between predictor variables were low (r < 0.70), indicating that multicollinearity was not a confounding factor in these models.

### 3.4. Best Subset Selection of the Predictive Model for Toxicity

Based on the previous results and including the parameter of potential clinical relevance, 15 parameters (BMI, gender, urea, GFR, neutrophils, emotional functioning, body image, LQ30 [GHS: “How would you rate your overall quality of life during the past week?”], social function, fatigue, LQ35 [Symptoms related to gastrointestinal tract: “Did you have abdominal pain?”], pain, appetite loss, cognitive function, and erythrocytes) were included in a best subset selection process. Best subset selection identified a model including BMI, gender, urea, neutrophils, emotional functioning, body image, LQ30, LQ35, and appetite loss, with an AUC of 0.709, as the best model (Final Model A). However, only BMI, gender, and emotional functioning had *p*-values < 0.1, whereas body image and LQ30 had *p*-values of 0.178 and 0.106, respectively. In the next three models, besides BMI, gender, and emotional functioning, we included body image plus LQ30 (Final Model B) and body image (Final Model C) or LQ30 (Final Model D) only. The model including LQ30 and body image achieved an AUC of 0.696, whereas the models with only LQ30 or body image achieved AUCs of 0.690. Finally, we tested the basic model, including only BMI, gender, and emotional functioning (Final Model E). This model showed a discrimination ability of 0.687. In all described models, correlations between predictor variables were low (r < 0.70), indicating that multicollinearity was not a confounding factor in these models (Table S5).

To finally decide which model to choose, we performed internal validation for all five final models by using internal cross validation and bootstrapping to assess the potential overfitting of these models. Even though the final model A showed the best discrimination ability, the AUC in the internal cross validation cohort and the adjusted AUC for overfitting were lower compared to the other final models. Therefore, we decided to use the basic model (Final Model E) as our prediction model because only in this model were all three variables significant, and this model had the highest AUC in the validation cohort of internal validation (Table S5, Figure S1).

### 3.5. Predictive Toxicity Model Using BMI, Gender, and Emotional Functioning

The risk score including BMI, gender, and emotional functioning was calculated, as described in detail in Supplementary Methods (Table 2).

Formula for calculating the risk score:

**−((1 − (((EmotionalPoints/4) − 1)/3)) × 100) − (BMI × 5) + Gender**

***[for male insert: 44, for female insert: 88].***

**Figure 1:**
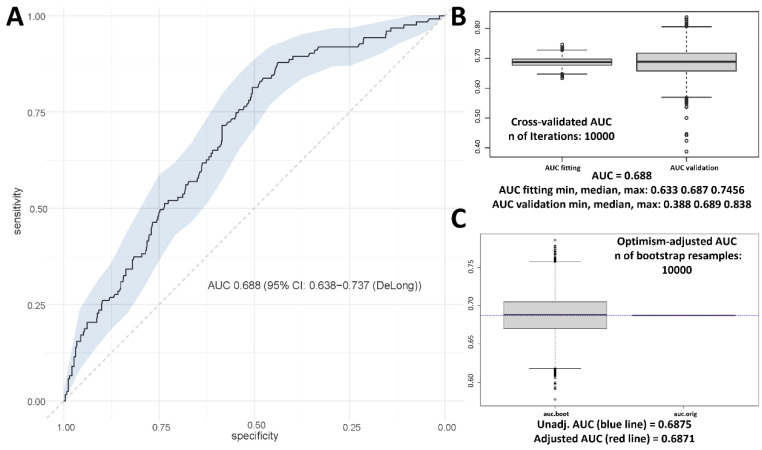
(**A**) Discrimination ability of the final model evaluated by calculating the AUC; (**B**) internal cross validation performed with the “modelvalid” function in R; (**C**) optimization-adjusted AUC calculated with the “aucadj” function in R.

**Figure 2:**
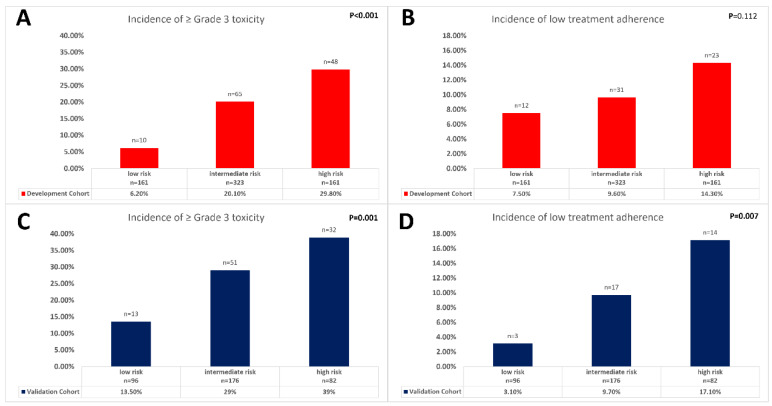
Distribution of toxicity and low treatment adherence within the three risk groups in (**A**,**C**) the development cohort, and (**B**,**D**) the validation cohort, tested using the chi-squared test.

The median score in the development cohort was −147.67 (range −243.17 to −5.50). The model achieved a discrimination of AUC 0.688 (95% CI: 0.638–0.737, Figure 1A) and goodness-of-fit *p*-value using the Hosmer–Lemeshow test was 0.100. Internal cross validation yielded a median AUC of 0.687 (0.633 to 0.746) for the fitting cohort and 0.689 (0.388–0.84) for the validation cohort (Figure 1B). Bootstrapping validation indicated a minor overfitting of 0.0004 and resulted in an adjusted AUC of 0.687 (Figure 1C). After dividing the development cohort into three toxicity risk groups, the odds of experiencing high-grade toxicity were 3.8 (95% CI, 1.898–7.626) times higher in the intermediate and 6.4 (3.111–13.225) times higher in the high-risk group. The incidence of high-grade toxicity was significantly different between the risk groups (*p* < 0.001, Figure 2A). The incidence of low treatment adherence was lowest for patients with low risk for toxicity (7.5%) and increased to 14.3% for patients with high risk for toxicity (*p* = 0.112, Figure 2B).

### 3.6. Development and Validation Cohort Characteristics

**Figure 3:**
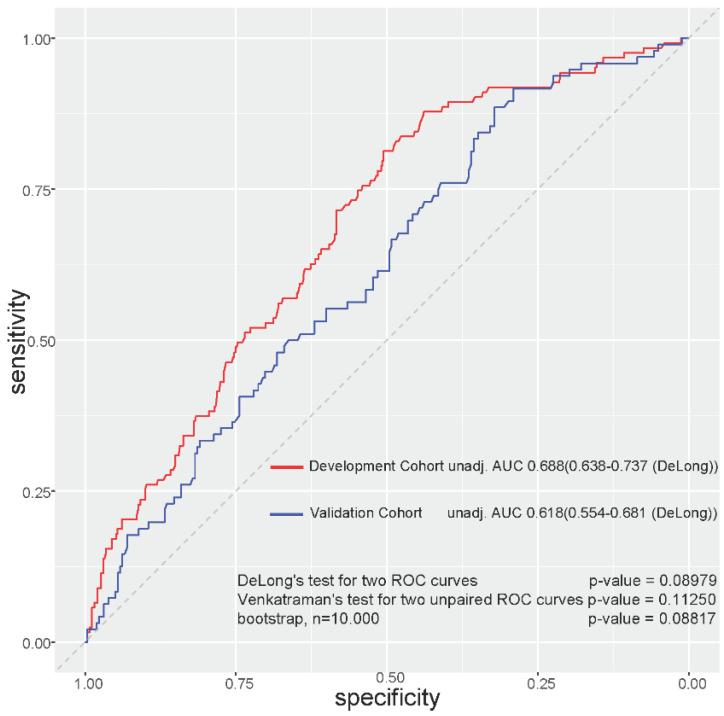
Discrimination ability of the final model in the development cohort and in the validation cohort. Discrimination ability between cohorts was compared using the DeLong nonparametric test, the Venkatraman test for two unpaired ROC curves, and by bootstrapping.

Table 3 includes patient characteristics for both the development and the validation cohorts. Significantly more patients in the development cohort were classified as ECOG grade 1 or 2 (*p* < 0.001), cT4 and cN+ tumors occurred more often in the validation cohort (*p* = 0.009, 0.049), and more patients in the validation cohort experienced high-grade toxicity during neoadjuvant CRT (*p* = 0.001), but the incidence of incomplete treatment adherence did not differ significantly between either cohort (11.8% vs. 10.1%). In the validation cohort, a higher risk score was statistically significantly associated with a higher incidence of toxicity (*p* = 0.001, Figure 2C) and lower treatment adherence (*p* = 0.007, Figure 2D). AUC for the predictive model was 0.618 (CI 95%, 0.554–0.681), and discrimination ability was not statistically different compared to the development cohort (DeLong’s test *p* = 0.09, Figure 3).

## 4. Discussion

Our analyses indicate that pretreatment risk assessment for high-grade toxicity during neoadjuvant CRT for rectal cancer based on gender, BMI, and emotional functioning may be useful to identify patients at higher risk for experiencing treatment-related toxicity. In both the development and validation cohorts, the three risk groups were associated with high-grade toxicity and treatment adherence. To our knowledge, this is the first predictive model for high-grade toxicity to CRT based on a large, randomized phase III trial cohort for rectal cancer.

The current development of multimodal treatment concepts in locally advanced rectal cancer, including the advent of intensified treatment such as TNT, can be associated with higher toxicity and reduced treatment adherence, posing new challenges for oncologists in deciding which therapy to recommend to each individual patient [10,28,29]. The higher risk for CRT-related toxicity in females can be explained, at least in part, by gender-specific differences in 5-FU metabolism or body fat proportions [30], whereas decreased physiologic reserve capacities, less metabolism resilience, limited fat stores, and decreased muscle proportion leading to different metabolisms and distributions of chemotherapeutics could explain the higher CRT-related toxicity in underweight patients [31]. A recent meta-analysis published by Holyoake et al. reported a significant association between dose and volume exposure of the small bowel and toxicity in rectal cancer. Toxicity seems to increase, not only with the absolute volume of the small bowel irradiated, but also in correlation with the relative differences in the volume irradiated with high doses. They proposed additional dose constraints for higher doses (e.g., V45Gy < 44 cm^3^), in addition to the QUANTEC recommendation of V15Gy < 20 cm^3^ to predict toxicity. However, no gender-specific analyses were carried out. The gender-specific correlation between dose and volume of exposure and toxicity should be further investigated. In addition, to use small bowel dose as a predictive factor for toxicity, guidelines for consistent contouring must be followed, and other aspects, e.g., the impact of small bowel movement, should, of course, be considered [32].

Intriguingly, in our analysis, the assessment of pretreatment physical and psychological burden also identified patients at higher risk for high-grade adverse events. Emotional functioning remains a significant contributor in the final prediction model and is one of the core domains of the EORTC QLQ-CR30 questionnaire. Tavoli et al. reported an association between emotional functioning and anxiety, specifically depression, in 137 gastrointestinal cancer patients [33]. Besides weak social functioning, which correlated with higher-grade toxicity in our development cohort, lack of family support can decrease individual coping capacities. This lack of social support can lead to higher morbidity, mortality, and delayed reporting of symptoms by patients, which could negatively impact treatment outcome [34]. Patients at increased risk of high-grade toxicity should be referred early to psycho-oncological counselling to support their psychological health [35].

Previous studies in older patients showed that in addition to geriatric assessments, chemotherapy drug/doses, baseline hemoglobin, creatinine clearance, and liver function predicted toxicity [25,26,27], which is partly consistent with our findings in univariable analysis, but did not remain significant after consideration of gender or in the further regression models (Table S6). Therefore, in our trial cohort, pretreatment blood parameters were not useful predictors for organ or bone marrow toxicity. Because of patient selection based on trial exclusion criteria, blood parameters may have a predictive ability in a cohort including patients with more serious comorbidities.

Our study has limitations. First, this work constitutes a post hoc analysis. Second, albeit greatly overlapping, the QLQ-CR-38 questionnaire has been replaced by the modified QLQ-CR-29 in patients with colorectal cancer. Third, we decided to categorize only patients with at least grade 3 adverse events in the high toxicity risk group; hence, lower-grade adverse events were not incorporated in our analyses. Fourth, baseline contoured planning CTs were not available for secondary analyses to address possible correlations between dose–volume exposure of organs at risk and toxicity. Therefore, dose–volume exposure of, e.g., the small bowel, is a potentially confounding factor which cannot be addressed in this analysis. It remains to be shown whether a model that incorporates dose exposure of the small bowel could improve discriminatory accuracy [32]. Fifth, for 221 patients (25.5%) in the development cohort, the toxicity prediction score could not be calculated because of missing values for the baseline parameter. Missing baseline patient-reported data is a potential confounding factor for toxicity analyses and could bias our results [36,37]. Sixth, the discrimination ability of our model narrowly fails to pass an AUC of 0.7, which was defined as the cut-off for acceptable discrimination by Hosmer and Lemeshow. Nevertheless, we believe that our model provides practically applicable information for physicians, and that our model can serve as a benchmark for further development of new predictive models [38]. Furthermore, only further studies will be able to shed light on the extent to which pre-therapeutic tests for DPD deficiency can reduce treatment-related side effects [39].

## 5. Conclusions

In summary, we have developed and validated a practical toxicity score based on gender, BMI, and emotional function. Our model can be useful for treating physicians to select patients who need more regular clinic visits, or who could benefit from early concomitant psycho-oncological counselling, as well as in aiding in the promotion of shared decision making with patients to determine the optimal individual treatment approach.

## Figures and Tables

**Table 1:** Association between baseline clinical characteristics and toxicity in the development cohort.

*Characteristics*	No.	Low-Grade Toxicity n = 676	High-Grade Toxicity n = 155	*p*-Value
***Percentage***		81.3%	18.7%	
***Treatment Arm***				
5-FU arm	420	347 (82.6%)	73 (17.4%)	
5-FU/Ox arm	408	326 (79.9%)	82 (20.1%)	0.316 *
***Age***				
continuous	831	676 (81.3%)	155 (18.7%)	0.749 **
(median age)		63.50 years	62.70 years	
***Gender***				
Male	590	501 (84.9%)	89 (15.1%)	
Female	241	175 (72.6%)	66 (27.4%)	<0.001 *
***ECOG***				
Grade 0	617	508 (82.3%)	109 (17.7%)	
Grade 1 + 2	202	159 (78.7%)	43 (21.3%)	0.251 *
***BMI***				
continuous	827	672 (81.3%)	155 (18.7%)	0.001 **
(median BMI)		26.75 kg/m^2^	25.3 kg/m^2^	
***cT-category***				
cT2	41	29 (70.7%)	12 (29.3%)	
cT3	736	605 (82.2%)	131 (17.8%)	
cT4	49	37 (75.5%)	12 (24.5%)	0.107 *
***cN-category***				
cN0	219	177 (80.8%)	42 (19.2%)	
cN1/cN2	593	482 (81.3%)	111 (18.7%)	0.882 *
***Grading***				
G1	49	43 (87.8%)	6 (12.2%)	
G2	658	531 (80.7%)	127 (19.3%)	
G3	70	62 (88.6%)	8 (11.4%)	0.145 *
***Tumor Localization***				
Low	308	247 (80.2%)	61 (19.8%)	
Middle/High	511	418 (81.8%)	93 (18.2%)	0.569 *

Correlations were assessed using the Pearson’s chi-squared test * or the Mann–Whitney U Test **. Abbreviations: No./n, number; 5-FU, 5-fluorouracil; Ox, oxaliplatin; ECOG, Eastern Cooperative Oncology Group; BMI, body mass index; cT, clinical tumor stage; cN, clinical node status, low, <0 cm to 5 cm of the anal verge; middle, 5 cm to 10 cm of the anal verge; high, >10 cm of the anal verge. Bold printed: significant *p* < 0.05.

**Table 2:** Multivariable binary logistic regression predictive model and scoring algorithms for the toxicity risk score.

Parameter	OR (95% CI)	Beta Coefficient	Response	Formula	Value
Gender	1.922 (1.259–2.932)	0.653	Male Female		44 points 88 points
***BMI*** (continuous)	0.933 (0.888–0.980)	−0.070	kg/m^2^	________ kg/m^2^ × 5	**-**_______points
***Emotional*** ***functioning***	0.985 (0.977–0.992)	−0.015			
		**Calculation as follows:**			
***Did you feel tense?***					
Not at All	A Little	Quite a Bit	Very Much	_____× 1	
1 point	2 points	3 points	4 points		
***Did you worry?***					
Not at All	A Little	Quite a Bit	Very Much	_____× 1	
1 point	2 points	3 points	4 points		
***Did you feel irritable?***					
Not at All	A Little	Quite a Bit	Very Much	_____× 1	
1 point	2 points	3 points	4 points		
***Did you feel depressed?***					
Not at All	A Little	Quite a Bit	Very Much	_____× 1	
1 point	2 points	3 points	4 points		
	**Formula to calculate points** **(Please insert the sum of the points according to your answers in the gray field of the formula) :**		$\left[1-\left(\frac{\left(\frac{\left[\_\_\_\_\_\_\right]}{4}-1\right)}{3}\right)\right]\times 100$		**-**______points
				**Sum**	**______points**
	**Risk Prediction**	**low** toxicity risk	**intermediate** toxicity risk	**high** toxicity risk	
		<−176.333 points	−176.333 to −118.083 points	>−118.083 points	

Abbreviations: BMI, body mass index; OR, odds ratio.

**Table 3:** Distributions of clinical characteristics and toxicity in the development and validation cohorts.

Characteristics	No.	Development Cohort n = 831	Validation Cohort n = 405	*p*-Value
***Percentage***		67.2%	32.8%	
***Treatment Arm***				
5-FU arm	625	420 (50.7%)	205 (50.7%)	
5-FU/Ox arm	607	408 (49.3%)	199 (49.3%)	0.995 *
***Age***				
continuous	1236	831 (67.2%)	405 (32.8%)	0.788 **
≤67.89 years	824	560 (32.0%)	264 (34.2%)	
>67.89 years	412	271 (68.0%)	141 (65.8%)	0.441 *
***Gender***				
Male	831	590 (71.0%)	284 (70.1%)	
Female	405	241 (29.0%)	121 (29.9%)	0.751 *
***ECOG***				
Grade 0	819	617 (75.3%)	341 (84.6%)	
Grade 1 + 2	403	202 (24.7%)	62 (15.4%)	<0.001 *
***BMI***				
continuous	1232	827 (67.1%)	405 (32.9%)	0.220 **
<20 kg/m^2^	52	37 (4.5%)	15 (3.7%)	
20–24.9 kg/m^2^	369	253 (30.6%)	116 (28.6%)	
25–26.9 kg/m^2^	234	159 (19.2%)	75 (18.5%)	
27–29.9 kg/m^2^	307	206 (24.9%)	101 (24.9%)	
≥30 kg/m^2^	270	172 (20.8%)	88 (24.2%)	0.693 *
***cT-category***				
cT2	54	41 (5.0%)	13 (3.2%)	
cT3	1086	736 (89.1%)	350 (86.4%)	
cT4	91	49 (5.9%)	42 (10.4%)	0.009 *
***cN-category***				
cN0	305	219 (27.0%)	86 (21.7%)	
cN1/cN2	903	593 (73.0%)	310 (78.3%)	0.049 *
Grading				
G1	64	49 (6.3%)	15 (3.9%)	
G2	998	658 (84.7%)	340 (88.5%)	
G3	99	70 (9.0%)	29 (7.6%)	0.152 *
***Tumor Localization***				
Low	465	308 (37.6%)	157 (39.0%)	
Middle/High	757	511 (62.4%)	246 (61.0%)	0.647 *
***Overall neoadjuvant treatment toxicity***				
Grade 0 + 1 + 2	971	676 (81.3%)	295 (72.8%)	
Grade 3 + 4	265	155 (18.7%)	110 (27.2%)	0.001 *
***Neoadjuvant Treatment Adherence***				
complete/nearly complete	1097	733 (88.2%)	364 (89.9%)	
incomplete	139	88 (11.8%)	41 (10.1%)	0.383 *

Correlations were assessed using the Pearson’s chi-squared test * or the Mann–Whitney U Test **. Complete neoadjuvant treatment adherence is defined as receiving the full doses of radiotherapy (50.4 Gy) and concurrent chemotherapy; nearly complete is defined as received 45 Gy or more of radiotherapy and 80% of concurrent chemotherapy; and incomplete is defined as receiving less than 45 Gy of radiotherapy or less than 80% of concurrent chemotherapy. Abbreviations: 5-FU, 5-fluorouracil; Ox, oxaliplatin; ECOG, Eastern Cooperative Oncology Group; BMI, body mass index; cT, clinical tumor stage; cN, clinical node status, low, <0 cm to 5 cm of the anal verge; middle, 5 cm to 10 cm of the anal verge; high, >10 cm of the anal verge. Bold printed: significant *p* < 0.05.

## Data Availability

The data presented in this study are not available due to data protection requirements of the CAO/ARO/AIO-04 trial.

## Supplementary Materials

The following supporting information can be downloaded at: https://www.mdpi.com/article/10.3390/cancers14184425/s1, Supplementary Methods; Table S1: Correlation of individual toxicity parameter and prediction model scoring in development cohort; Table S2: Association between baseline blood parameters and toxicity in the development cohort; Table S3: Association between baseline quality of life questions and toxicity in the development cohort; Table S4: Association between baseline global health status, functional scores, symptom scores/items, and toxicity in the development cohort; Table S5: Evaluations of predictive binary logistic regression models in the development cohort; Table S6: Association between baseline erythrocyte levels and glomerular filtration rate in all patients, in male and female patients, and toxicity in the development cohort; Figure S1: (**A**) Internal cross validation performed with the “modelvalid” function in R for Final Model A, (**B**) optimization-adjusted AUC calculated with the “aucadj” function in R for Final Model A, (**C**) internal cross validation performed with the “modelvalid” function in R for Final Model B, (**D**) optimization-adjusted AUC calculated with the “aucadj” function in R for Final Model B, (**E**) internal cross validation performed with the “modelvalid” function in R for Final Model C, (**F**) optimization-adjusted AUC calculated with the “aucadj” function in R for Final Model C, (**G**) internal cross validation performed with the “modelvalid” function in R for Final Model D, (**H**) optimization-adjusted AUC calculated with the “aucadj” function in R for Final Model D, (**I**) internal cross validation performed with the “modelvalid” function in R for Final Model E, (**J**) optimization-adjusted AUC calculated with the “aucadj” function in R for Final Model E.
