# Supplementary material for: Development and Validation of a Predictive Model for Toxicity of Neoadjuvant Chemoradiotherapy in Rectal Cancer in the CAO/ARO/AIO-04 Phase III Trial
Source: Cancers (Basel). 2022 Sep 12;14(18):4425. doi: 10.3390/cancers14184425 (PMC9497244; doi:10.3390/cancers14184425)
Supplement: Supplementary file 1 [file cancers-14-04425-s001.zip › cancers-1875244-supplementary.pdf]

Supplemental Data

1. **Supplementary Methods** Page: 02
2. **Table S1.** Correlation of individual toxicity parameter and prediction model scoring in development cohort Page: 03
3. **Table S2.** Association between baseline blood parameter and toxicity in development cohort Page:04
4. **Table S3.** Association between baseline quality of life questions and toxicity in development cohort Page: 05/06
5. **Table S4.** Association between baseline global health status, functional scales, symptom sales/items and toxicity in development cohort Page: 07/08
6. **Table S5.** Evaluations of predictive binary logistic regression models in development cohort Page: 09/10
7. **Table S6.** Association between baseline erythrocytes levels and glomerular filtration rate in all patients, in male and female patients and toxicity in development cohort Page: 11
8. **Supplementary Figure Legend** Page: 12
9. **Figure S1** Page: 13

Supplementary Methods

Development and validation of the predictive model

Using a best subset selection approach, the best predictive binary multivariable regression models according to AIC (Akaike Information Criterion) were calculated for clinicopathologic characteristics, pretreatment peripheral blood parameter and baseline results of QLQ including parameter with P<0.1 in the association testing or parameter deemed to be of clinical interest by physicians' decision [25-27]. The specifications of the statistical procedure restricted the number of variables in each model to 15 or less. Finally, parameters with P<0.1 in regression models respectively parameters by physicians' decisions were selected for the final models. Internal Validation was performed for all final models using internal cross validation and bootstrapping to analyze potential overfitting of the model. For internal cross validation the "modelvalid" function in R with 10.000 iterations and to calculate an optimism adjusted AUC the "aucadj" function in R with 10.000 resamples were used. Based on the results of the internal validation and the previous results the authors have chosen the final model.

We calculated an absolute point value for each variable in the final model by dividing the beta coefficient of the variables by the lowest beta coefficient, rounding to the nearest whole number, and summing all point values [25-27]. We evaluated the discrimination ability of the model by calculating the area under the curve (AUC) and goodness-of-fit was assessed using the Hosmer-Lemeshow-Test. Next, we divided the cohort into three toxicity risk groups: low, intermediate, and high risk, with the lower quartile of the continuous risk score defined as low risk, the two intermediate quartiles defined as intermediate risk, and the upper quartile defined as high risk. The distribution of toxicity within the three risk groups was tested using the chi-squared test.

For external validation, we assessed the discriminatory ability of the model in the validation cohort by calculating the AUC and evaluating the distribution of high-grade toxicity within the three risk groups using the chi-squared test. The discriminative ability in the validation cohort was compared with the development cohort using the Delong nonparametric test, the Venatramans test for two unpaired ROC curves, and by bootstrapping. In addition, we analyzed the association between toxicity risk groups and treatment adherence and specific reported adverse events using chi-squared tests.

Table S1. Correlation of individual toxicity parameter and prediction model scoring in development cohort

Parameter[#]	High Grade Toxicity	P-Value*	Percentage incorrect classified[#]	Low risk**	Percentage correct classified[#]	High risk***
Vomiting	6	**<0.001**	0.00%	0//4	100.00%	4//4
Hand Foot Syndrome	2	0.463	0.00%	0//1	100.00%	1//1
Motor disorder	1	0.238	0.00%	0//1	100.00%	1//1
Dyspnea	3	0.157	0.00%	0//3	66.67%	2//3
Fatigue	10	**<0.001**	0.00%	0//7	57.14%	4//7
Pain	23	**<0.001**	0.00%	0//19	52.63%	10//19
Leucopenia	12	**0.004**	0.00%	0//9	55.56%	5//9
Arrythmia	3	0.275	0.00%	0//2	50.00%	1//2
Rectal bleeding	8	0. 322	0.00%	0//7	42.86%	3//7
Anemia	6	0.067	0.00%	0//3	33.33%	1//3
Kidney failure	4	0.655	0.00%	0//3	33.33%	1//3
Mucositis	15	**0.007**	0.00%	0//12	33.33%	4//12
Pollakiuria	6	0.793	0.00%	0//5	20.00%	1//5
Radiation Dermatitis	14	**0.032**	0.00%	0//11	27.27%	3//11
Constipation	1	0.576	0.00%	0//1	0.00%	0//1
Cystitis	2	0.692	0.00%	0//1	0.00%	0//1
Diarrhea	67	**<0.001**	5.66%	3//53	52.83%	28//53
Nausea	11	**<0.001**	11.11%	1//9	55.55%	5//9
Proctitis	10	0.424	16.67%	1//6	33.33%	2//6
Elevated Transaminase	9	**0.002**	16.67%	1//6	66.67%	4//6
Infection	17	0.360	17.64%	3//17	29.41%	5//17
Cardiac Ischemia	2	0.732	50.00%	1//2	0.00%	0//2
Dysesthesia	0	0.213	n.c.	0//0	n.c.	0//0
Thrombopenia	3	0.508	n.c.	0//0	n.c.	0//0
Allergic Reaction	0	**0.005**	n.c.	0//0	n.c.	0//0
Alopecia	0	0.284	n.c.	0//0	n.c.	0//0
Fewer	0	0.663	n.c.	0//0	n.c.	0//0
Alkaline phosphatase	0	**0.001**	n.c.	0//0	n.c.	0//0
Bilirubin	0	0.834	n.c.	0//0	n.c.	0//0

[#] Ordered by percentage of misclassification of high-grade toxicity

* Correlations between risk groups and toxicity parameter Grade 0 vs 1 vs 2 vs 3 vs 4 were assessed using the Pearson's chi-squared test

**X//X [cases classified in low-risk group//all cases for who risk score was calculable]

***X//X [cases classified in high-risk group// all cases for who risk score was calculable]

Abbreviations: n.c.; not calculable

Table S2. Association between baseline blood parameter and toxicity in development cohort

**Blood parameter**	**No.**	**Average level** in development cohort	**low grade toxicity n = 676** **No.**	**Average level** low grade toxicity	**high grade toxicity n = 155** **No.**	**Average level** high grade toxicity	**P-value**	**AUC*** **
**Albumin** [g/dl]	634	4.18	512	4.18	122	4.16	0.724**	0.490
**Alkaline phosphatase** [U/l]	797	77.07	648	76.59	149	79.14	0.090**	**0.545**
**Bilirubin** [U/l]	807	0.51	655	0.52	152	0.49	0.115**	**0.459**
**Calcium** [mmol/l]	766	2.34	623	2.34	143	2.33	0.203**	0.466
**CEA** [ng/ml]	746	11.96	604	12.78	142	8.47	0.283**	0.471
**Protein** [g/dl]	727	7.21	585	7.20	142	7.25	0.394**	0.523
**Erythrocytes** [/pl]	829	4.66	674	4.69	155	4.51	**0.003****	**0.424**
**GOT** [U/l]	785	23.53	638	23.53	147	23.51	0.773**	0.508
**GPT** [U/l]	809	25.19	655	25.24	154	24.96	0.926**	0.498
**Hb** [g/dl]	830	13.60	675	13.66	155	13.34	0.094**	**0.457**
**Urea** [mg/dl]	742	25.05	599	25.10	143	24.84	0.881**	0.504
**Potassium** [mmol/dl]	829	4.26	674	4.26	155	4.26	0.765**	0.492
**Creatinine** [mg/dl]	826	0.88	671	0.89	155	0.86	0.150**	0.463
**GFR** [ml/min]	822	96.68	667	97.93	155	91.32	**0.017****	**0.438**
**LDH** [U/l]	740	185.55	599	185.62	141	185.23	0.783**	0.507
**Leukocytes** [/nl]	830	7.71	675	7.73	155	7.62	0.874**	0.496
**Sodium** [mmol/l]	828	139.90	673	139.94	155	139.74	0.693**	0.490
**Neutrophils** [/nl]	663	5.07	539	5.01	124	5.30	0.143**	**0.542**
**Platelets** [/nl]	829	285.31	675	284.88	154	287.20	0.909**	0.497

Correlations were assessed using the Mann-Whitney-U-Test**

*** positive value for the state variable = high grade toxicity

Cockcroft-Gault equation used as method of estimating GFR *[CCr={((140–age) x weight)/(72xSCr)}x 0.85 (if female);*

Abreviation in formular: CCr (creatinine clearance) = mL/minute; Age = years; Weight = kg; SCr (serum creatinine) = mg/dL]

Abbreviations: No./n, number; CEA, carcinoembryonic antigen; GOT, aspartate transaminase; GPT, alanine transaminase;

Hb, haemoglobin; GFR, glomerular filtration rate; LDH, lactate dehydrogenase;

Bold printed: significant **P<0.05** or **AUC > 0.54, < 0.46**

Table S3. Association between baseline quality of life questions and toxicity in development cohort

**EORTC QLQ-CR30**<br>**EORTC QLQ – CR38**	**No.**	**Average Score**<br>in development cohort	**low grade toxicity n = 676**<br>**No.**	**Average Score**<br>low grade toxicity	**high grade toxicity n = 155**<br>**No.**	**Average Score**<br>high grade toxicity	**P-value**	**AUC**
**LQ1 [Physical functioning]**	661	1.69	536	1.65	125	1.86	**0.031****	**0.556**
**LQ2 [Physical functioning]**	665	1.59	540	1.57	125	1.67	0.345**	0.523
**LQ3 [Physical functioning]**	663	1.20	538	1.18	125	1.32	0.077**	0.531
**LQ4 [Physical functioning]**	660	1.31	537	1.28	123	1.43	**0.040****	**0.544**
**LQ5 [Physical functioning]**	664	1.03	539	1.03	125	1.04	0.348**	0.507
**LQ6 [Role functioning]**	660	1.55	536	1.53	124	1.65	0.233**	0.529
**LQ7 [Role functioning]**	650	1.52	529	1.49	121	1.61	0.233**	0.529
**LQ8 [Dyspnoea]**	657	1.28	532	1.27	125	1.33	0.415**	0.517
**LQ9 [Pain]**	658	1.55	533	1.51	125	1.71	0.068**	**0.545**
**LQ10 [Fatigue]**	657	1.66	532	1.61	125	1.87	**0.005****	**0.574**
**LQ11 [Insomnia]**	661	1.80	536	1.76	125	1.97	**0.035****	**0.556**
**LQ12 [Fatigue]**	661	1.62	536	1.57	125	1.88	**0.002****	**0.581**
**LQ13 [Appetite loss]**	662	1.34	538	1.31	124	1.48	**0.049****	**0.542**
**LQ14 [Nausea and vomiting]**	661	1.13	537	1.12	124	1.19	0.177**	0.521
**LQ15 [Nausea and vomiting]**	660	1.03	535	1.02	125	1.06	0.086**	0.510
**LQ16 [Constipation]**	642	1.41	520	1.38	122	1.49	0.500**	0.515
**LQ17 [Constipation]**	642	1.92	521	1.90	121	1.99	0.389**	0.524
**LQ18 [Fatigue]**	645	1.77	523	1.72	122	2.02	**0.001****	**0.589**
**LQ19 [Pain]**	646	1.44	525	1.39	121	1.66	**0.005****	**0.566**
**LQ20 [Cognitive functioning]**	647	1.31	524	1.27	123	1.47	**0.002****	**0.566**
**LQ21 [Emotional functioning]**	642	1.80	521	1.74	121	2.08	**<0.001****	**0.609**
**LQ22 [Emotional functioning]**	646	2.58	524	2.50	122	2.94	**<0.001****	**0.617**
**LQ23 [Emotional functioning]**	642	1.67	519	1.61	123	1.89	**<0.001****	**0.591**
**LQ24 [Emotional functioning]**	638	1.88	516	1.81	122	2.16	**<0.001****	**0.608**
**LQ25 [Cognitive functioning]**	643	1.34	521	1.34	122	1.34	0.913**	0.502
**LQ26 [Social functioning]**	645	1.69	522	1.64	123	1.92	**0.004****	**0.575**
**LQ27 [Social functioning]**	642	1.63	521	1.59	121	1.81	**0.019****	**0.560**
**LQ28 [Constipation]**	636	1.47	518	1.46	118	1.52	0.348**	0.522
**LQ29 [Global health status]**	643	4.70	520	4.77	123	4.42	**0.012****	**0.429**
**LQ30 [Global health status]**	640	4.68	517	4.77	123	4.31	**<0.001****	**0.398**
**LQ31 [Micturition problems]**	649	2.13	524	2.15	125	2.06	0.359**	0.475
**LQ32 [Micturition problems]**	650	1.98	525	1.98	125	2.02	0.665**	0.512
**LQ33 [Micturition problems]**	654	1.12	529	1.11	125	1.14	0.870**	0.502
**LQ34 [Symptom's gastro- intestinal tract]**	651	1.73	528	1.71	123	1.85	0.307**	0.527
**LQ35 [Symptom's gastro- intestinal tract]**	652	1.38	528	1.34	124	1.56	**0.011****	**0.557**
**LQ36 [Symptom's gastro- intestinal tract]**	651	1.64	527	1.64	124	1.64	0.871**	0.496
**LQ37 [Symptom's gastro- intestinal tract]**	653	1.92	529	1.90	124	1.99	0.419**	0.522
**LQ38 [Symptom's gastro- intestinal tract]**	653	1.43	528	1.42	125	1.49	0.702**	0.509
**LQ39 [Weight loss]**	653	1.77	529	1.74	124	1.90	0.150**	0.538
**LQ40 [Chemotherapy side effects]**	647	1.50	524	1.48	123	1.60	0.363**	0.522
**LQ41 [Chemotherapy side effects]**	647	1.11	524	1.11	123	1.14	0.450**	0.511
**LQ42 [Chemotherapy side effects]**	655	1.19	531	1.18	124	1.23	0.142**	0.526

**LQ43 [Body image]**	642	1.40	518	1.37	124	1.51	**0.037****	**0.547**
**LQ44 [Body image]**	640	1.24	518	1.23	122	1.26	0.641**	0.509
**LQ45 [Body image]**	641	1.63	520	1.61	121	1.72	0.190**	0.534
**LQ46 [Future Perspective]**	653	3.03	529	3.00	124	3.19	0.053**	**0.553**
**LQ47 [Sexual functioning]**	595	2.15	484	2.18	111	2.02	0.105**	**0.453**
**LQ48 [Sexual functioning]**	587	1.99	478	2.02	109	1.89	0.170**	**0.460**
**LQ49 [Sexual enjoyment]**	310	2.92	259	2.92	51	2.90	0.941**	0.497
**LQ50 [Male sexual problem]**	377	2.04	327	2.03	50	2.10	0.840**	0.508
**LQ51 [Male sexual problem]**	360	1.77	312	1.76	48	1.85	0.786**	0.511
**LQ52 [Female sexual problems]**	70	1.60	51	1.45	19	2.00	**0.033****	**0.640**
**LQ53 [Female sexual problems]**	69	1.26	51	1.22	18	1.39	0.261**	**0.561**
**LQ54 [Stoma]**								
**yes**	108		88	81.5%	20	18.5%		
**no**	440		361	82.0%	79	18.0%	0.826*	
**LQ55 [Defecation problems]**	489	2.62	395	2.65	94	2.52	0.242**	0.463
**LQ56 [Defecation problems]**	495	1.57	401	1.58	94	1.54	0.626**	0.486
**LQ57 [Defecation problems]**	490	2.17	396	2.17	94	2.14	0.680**	0.487
**LQ58 [Defecation problems]**	489	1.34	395	1.33	94	1.39	0.391**	0.521
**LQ59 [Defecation problems]**	485	2.38	393	2.36	92	2.47	0.322**	0.532
**LQ60 [Defecation problems]**	489	1.60	396	1.61	93	1.59	0.728**	0.490
**LQ61 [Defecation problems]**	494	1.48	401	1.45	93	1.58	0.394**	0.523
**LQ62 [Stoma-related problems]**	129	2.22	104	2.12	25	2.68	**0.031****	**0.634**
**LQ63 [Stoma-related problems]**	129	2.26	104	2.17	25	2.64	0.111**	**0.599**
**LQ64 [Stoma-related problems]**	129	2.49	103	2.46	25	2.64	0.479**	**0.544**
**LQ65 [Stoma-related problems]**	128	1.80	99	1.81	23	1.78	0.885**	0.509
**LQ66 [Stoma-related problems]**	122	1.73	100	1.72	24	1.79	0.775**	0.517
**LQ67 [Stoma-related problems]**	124	2.33	102	2.25	24	2.67	0.129**	**0.596**
**LQ68 [Stoma-related problems]**	126	2.40	102	2.33	24	2.67	0.188**	**0.584**

Correlations were assessed using the Pearson's chi-squared test* or Mann-Whitney-U-Test**

Abbreviations: No./n, number; LQ, quality of life, EORTC; European Organisation for Research and Treatment of Cancer
Bold printed: significant P<0.05 or **AUC ≥ 0.540 / ≤ 0.460**

Table S4. Association between baseline global health status, functional scales, symptom sales/items and toxicity in development cohort

EORTC QLQ-C30<br>EORTC QLQ – CR38<br>[integrated Questions]	No.	Average Score<br>in development cohort	low grade toxicity<br>n = 676<br>No.	Average Score<br>low grade toxicity	high grade toxicity<br>n = 155<br>No.	Average Score<br>high grade toxicity	P-value	AUC
Physical functioning [1-5]	657	88.08	534	88.77	123	85.09	0.107**	**0.456**
Physical functioning° [1-5]	664	87.91	539	88.72	125	84.40	0.066**	**0.450**
Role functioning [6,7]	649	82.43	528	83.11	121	79.48	0.316**	0.474
Role functioning° [6,7]	661	81.95	537	82.71	124	78.63	0.235**	0.470
Emotional functioning [21-24]	624	67.36	505	69.51	119	58.26	<0.001**	**0.358**
Emotional functioning° [21-24]	649	67.20	526	69.46	123	57.54	<0.001**	**0.352**
Cognitive functioning [20,25]	641	89.31	519	89.92	122	86.75	0.064**	**0.453**
Cognitive functioning° [20,25]	649	89.09	526	89.73	123	86.31	0.052**	**0.451**
Social functioning [26,27]	639	78.04	518	79.67	121	71.07	0.005**	**0.423**
Social functioning° [26,27]	648	77.88	525	79.43	123	71.27	0.007**	**0.427**
Fatigue [10,12,18]	637	22.57	515	20.67	122	30.60	0.001**	**0.595**
Fatigue° [10,12,18]	662	22.85	537	21.00	125	30.80	0.007**	**0.592**
Nausea [14,15]	657	2.59	533	2.22	124	4.17	0.188**	0.520
Nausea° [14,15]	664	2.61	539	2.26	125	4.13	0.210**	0.519
Pain [9,19]	641	16.28	520	14.78	121	22.73	0.027**	**0.557**
Pain° [9,19]	663	16.74	538	15.27	125	23.07	0.030**	**0.556**
Dyspnoea [8]	657	9.23	532	8.83	125	10.93	0.415**	0.517
Insomnia [11]	661	26.78	536	25.50	125	32.27	0.035**	**0.556**
Appetite loss [13]	662	11.43	538	10.41	124	15.86	0.049**	**0.542**
Constipation [16]	642	13.50	520	12.82	122	16.39	0.500**	0.515
Diarrhoea [17]	642	30.63	521	30.07	121	33.06	0.389**	0.524
Financial difficulties [28]	636	15.67	518	15.32	118	17.23	0.348**	0.522
Global Health Status [29-30]	640	61.60	517	62.91	123	56.10	0.001**	**0.407**
Global Health Status° [29-30]	643	61.42	520	62.67	123	56.10	0.002**	**0.410**
Micturition problems [31-33]	644	24.83	519	24.83	125	24.80	0.724**	0.490
Micturition problems° [31-33]	653	24.74	528	24.73	125	24.80	0.763**	0.492
Symptoms related to gastrointestinal tract [34-38]	636	20.63	513	20.04	123	23.09	0.448**	0.522
Symptoms related to gastrointestinal tract° [34-38]	655	20.31	531	19.60	124	23.33	0.259**	0.532
Weight loss [39]	653	25.83	529	24.83	124	30.11	0.150**	0.538
Chemotherapy Side Effects [40-42]	637	8.72	517	8.36	120	10.28	0.321**	0.526
Chemotherapy Side Effects° [40-42	655	9.02	530	8.53	125	11.07	0.238**	0.530
Body image [43-45]	626	86.17	508	86.57	118	84.46	0.105**	**0.456**
Body image° [43-45]	646	85.85	522	86.55	124	82.93	0.029**	**0.442**
Future Perspective [46]	653	32.21	529	33.46	124	26.88	0.053**	**0.447**
Sexual functioning [47-48]	586	64.31	477	63.49	109	67.89	0.154**	**0.543**
Sexual functioning° [47-48]	596	64.29	485	63.33	111	68.47	0.091**	**0.550**
Sexual Enjoyment [49]	310	36.02	259	35.91	51	36.60	0.941**	0.503
Male Sexual Problems [50-51]	360	30.00	312	29.65	48	32.29	0.966**	0.502
Male Sexual Problems° [50-51]	377	30.59	327	30.22	50	33.00	0.950**	0.503
Female Sexual Problems [52-53]	69	14.01	51	11.11	18	22.22	0.080**	**0.618**
Female Sexual Problems° [52-53]	70	14.05	51	11.11	19	21.93	0.061**	**0.625**
Defecation Problems [55-61]	479	29.06	385	29.00	94	29.28	0.992**	0.500

**Defecation Problems° [55-61]**	495	28.60	401	28.44	94	29.28	0.815**	0.508
**Stoma related problems [62-68]**	117	38.34	95	37.09	22	43.72	0.333**	**0.566**
**Stoma related problems° [62-68]**	128	39.54	103	37.39	25	48.37	0.090**	**0.609**

Correlations were assessed using the Pearson's chi-squared test* or
Mann-Whitney-U-Test**

° Patients integrated which answer at least 50% of necessary questions to calculate multi-item scale.

Calculation following EORTC recommendations

Abbreviations: No./n, number; LQ, quality of life; EORTC; European Organisation for Research and Treatment of Cancer

Bold printed: significant P<0.05 or **AUC ≥ 0.540 / ≤ 0.460**

Table S5. Evaluations of predictive binary logistic regression models in development cohort

Model	No.	Parameter (Coefficients:)	P-value*	AIC**	AUC***
**Clinical Characteristics**	743 (missing n=88)	Intercept (-0.82355608)	0.420		
Best Subset Selection[1]		Treatment Arm (0.16716386) [#]	0.390		
Hosmer-Lemeshow-Test P=0.340		Gender (0.81495441) [#]	**<0.001**		
		ECOG (0.06411142) [#]	0.776		
		BMI -0.05125578) [#]	**0.030**		
		cN (-0.03648509) [#]	0.868		
		Grading (-0.14491513) [#]	0.562		
		Localisation (-0.19623826) [#]	0.325	697.5	0.629 (0.574-0.685)
**Clinical Characteristics**	827 (missing n=4)	Intercept (-0.94506)	0.155		
Best Subset Selection of		BMI (-0.05465) [#]	**0.012**		
selective parameters[2]		Gender (0.69088) [#]	**<0.001**		
Hosmer-Lemeshow-Test P=0.987				781.3	0.619 (0.569-0.670)
**Baseline blood parameter**	467 (missing n=364)	Intercept (0.216769500)	0.913		
Best Subset Selection[3]		Albumin (-0.114188462) [#]	0.565		
Hosmer-Lemeshow-Test P=0.818		Bilirubin (-0.515599423) [#]	0.292		
		CEA (-0.003118283) [#]	0.608		
		Protein (0.177095325) [#]	0.436		
		Erythrocytes (-0.455139398) [#]	**0.098**		
		GOT (0.008121155) [#]	0.704		
		GPT (0.005171733) [#]	0.658		
		Urea (0.022441518) [#]	**0.066**		
		GFR (-0.010193128) [#]	**0.043**		
		LDH (-0.001274742) [#]	0.646		
		Neutrophils (0.124784691) [#]	**0.098**		
		Platelets (-0.001131022) [#]	0.422	484.0	0.636 (0.573-0.700)
**Baseline blood parameter**	605 (missing n=226)	Intercept (0.2764754)	**0.005**		
Best Subset Selection of		Erythrocytes (-0.0097520) [#]	0.262		
selective parameters[4]		Urea (0.0005902) [#]	0.709		
Hosmer-Lemeshow-Test P=0.313		GFR (-0.0015014) [#]	**0.016**		
		Neutrophils (0.0171176) [#]	**0.057**	585.8	0.598 (0.539-0.656)
**Baseline quality of life**	550 (missing n=281)	Intercept (-1.64547415)	**0.045**		
**Single questions**		LQ04 (0.05773877) [#]	0.765		
Best Subset Selection[5]		LQ18 (0.02976547) [#]	0.852		
Hosmer-Lemeshow-Test P=0.243		LQ19 (0.17398940) [#]	0.286		
		LQ21 (-0.10305380) [#]	0.514		
		LQ22 (0.42112661) [#]	**0.001**		
		LQ30 (-0.18142651) [#]	**0.077**		
		LQ34 (-0.03627589) [#]	0.778		
		LQ43 (-0.12063575) [#]	0.445		
		LQ47 (-0.07342751) [#]	0.567	516.7	0.655 (0.597-0.713)
**Baseline quality of life items and scales**	567 (missing n=264)	Intercept (-2.080081517)	0.004		
Best Subset Selection[6]		Emotional functioning (-0.015630908) [#]	**0.003**		
Hosmer-Lemeshow-Test P=0.145		Fatigue (0.007650370) [#]	0.140		
		Diarrhoea (-0.002382707) [#]	0.498		
		Global Health Status (0.008998047) [#]	0.176		
		Body image (0.011655310) [#]	**0.051**		
		Sexual functioning (0.001986052) [#]	0.682	533.9	0.668 (0.611-0.724)
**Baseline quality of life items and scales**	623 (missing n=208)	Intercept (0.14800000)	0.402		
Best Subset Selection of		Physical functioning (-0.00046560)	0.716		
selective parameters[7]		Emotional functioning (-0.00247000) [#]	**0.007**		
Hosmer-Lemeshow-Test P=0.580		Cognitive functioning (0.00093010)	0.397		
		Social functioning (-0.00014180) [#]	0.854		
		Fatigue (0.00133400) [#]	0.199		
		Pain (0.00073910) [#]	0.353		
		Appetite loss (-0.00001063) [#]	0.989		
		Global Health Status (0.00062900) [#]	0.501		
		Body image (0.00123700) [#]	0.181		
		Future perspective (0.00019810) [#]	0.732	601.4	0.623 (0.570-0.677)
**Final Model A**	469 (missing n=362)	Intercept (-0.926601273)	0.447		
Best Subset selection		BMI (-0.054678961) [#]	**0.062**		
"Based on previous results"[8]		Gender (0.706672622) [#]	**0.005**		
Hosmer-Lemeshow-Test P=0.234		Urea (0.008031763) [#]	0.504		
		Neutrophils (0.092355330) [#]	0.173		
		Emotional functioning (-0.013418757) [#]	**0.016**		
		Body image (0.008973951) [#]	0.178		
		LQ30 (-0.177160805) [#]	0.106		
		LQ35 (0.183168361) [#]	0.259		
		Appetit loss (-0.002659745) [#]	0.611	453.8	0.709 (0.655-0.764)
**Final Model B**	619 (missing n=212)	Intercept (0.588258740)	0.499		
Best Subset Selection of		BMI (-0.072152305) [#]	**0.005**		
selective parameters[9]		Gender (0.646956793) [#]	**0.003**		
Hosmer-Lemeshow-Test P=0.202		Emotional functioning (-0.013920157) [#]	**0.004**		
		Body image (0.007435091) [#]	**0.188**		
		LQ30 (-0.159156367) [#]	**0.081**	580.2	0.696 (0.648-0.746)
**Final Model C**	619 (missing n=212)	Intercept (0.253812032)	0.765		
Best Subset Selection of selective		BMI (-0.071281836) [#]	**0.006**		
parameters by physicians decision[12]		Gender (0.667591130) [#]	**0.002**		
Hosmer-Lemeshow-Test P=0.056		Emotional functioning (-0.017094477) [#]	**<0.001**		
		Body image (0.004426138) [#]	0.407	584.1	0.690 (0.640-0.740)

**Final Model D**<br>Best Subset Selection of selective parameters by physicians decision[11]<br>Hosmer-Lemeshow-Test P=0.151	619 (missing n=212)	Intercept (0.82552802) #	0.324		
		BMI (-0.07036213) #	**0.006**		
		Gender (0.63487046) #	**0.003**		
		Emotional functioning (-0.01249614) #	**0.006**		
		LQ30 (-0.10181934) #	0.232	591.9	0.690 (0.641-0.739)
**Final Model E**<br>Best Subset Selection of selective parameters by physicians decision[12]<br>Hosmer-Lemeshow-Test P=0.100	619 (missing n=212)	Intercept (0.48336350)	0.544		
		BMI (-0.06952360) #	**0.006**		
		Gender (0.65310730) #	**0.002**		
		Emotional functioning (-0.01544728) #	**<0.001**	595.1	0.687 (0.638-0.737)

*P-value in specific binary logistic regression model
**calculated AIC of binary logistic regression model
***calculated AUC (95% confidence interval) of binary logistic regression model
Correlations between predictor variables were low (r < .70), indicating that multicollinearity was not a confounding factor in this model

[1] Parameter included in Best-Subset Selection modelling: treatment arm, age, gender, ECOG, BMI, cT, cN, grading, tumor localisation
[2] Parameter included in Best-Subset Selection modelling: gender, BMI
[3] Parameter included in Best-Subset Selection modelling: albumin, alkaline phosphatase, bilirubin, calcium, CEA, protein, erythrocytes, GOT, GPT, b, urea, GFR, LDH, neutrophils, platelets
Parameter not included by physicians' decision: potassium, sodium – no hint of association with toxicity tested by Mann-Whitney-U-Test or AUC.
Leukocytes not included because neutrophils are subgroup of leukocytes. Creatine not included as parameter was used to calculate GFR based on Cockcroft-Gault-formular.
[4] Parameter included in Best-Subset Selection modelling: erythrocytes, urea, GFR, neutrophils
[5] Parameter included in Best-Subset Selection modelling: LQ01, LQ04, LQ11, LQ13, LQ18, LQ19, LQ20, LQ21, LQ22, LQ26, LQ30, LQ34, LQ43, LQ46, LQ47
[6] Parameter included in Best-Subset Selection modelling: physical functioning, emotional functioning, cognitive functioning, social functioning, fatigue, pain, insomnia, appetite loss, constipation, diarrhoea, global health status, symptoms related to gastrointestinal tract, body image, future perspective, sexual functioning
[7] Parameter included in Best-Subset Selection modelling by physicians' decision: physical functioning, emotional functioning, cognitive functioning, social functioning, fatigue, pain, appetite loss, global health status, body image, future perspective
[8]Parameter included in Best-Subset Selection modelling based on the results of the previous models: BMI, gender, urea, GFR, neutrophils, emotional functioning, body image, LQ30.
Additional added by Physician decision: social functioning, fatigue, LQ35, pain, appetite loss, cognitive functioning, erythrocytes
[9] Parameter included in Best-Subset Selection modelling by physicians' decision: BMI, gender, emotional functioning, body image, LQ30
[10] Parameter included in Best-Subset Selection modelling by physicians' decision: BMI, gender, emotional functioning, body image
[11] Parameter included in Best-Subset Selection modelling by physicians' decision: BMI, gender, emotional functioning, LQ30
[12] Parameter included in Best-Subset Selection modelling by physicians' decision: BMI, gender, emotional functioning

Abbreviations: No, number; AUC, areas under the curve
Bold printed: significant **P<0.05,** Bold printed and underlined **P≤0.10**

Table S6. Association between baseline erythrocytes levels and glomerular filtration rate in all patients, in male and female patients and toxicity in development cohort

	Blood parameter	No.	**Average level** in development cohort	**low grade toxicity n = 676 No.**	**Average level** low grade toxicity	**high grade toxicity n = 155 No.**	**Average level** high grade toxicity	P-value	AUC***
**All Patients**	**Erythrocytes** [/pl]	829	4.66	674	4.69	155	4.51	**0.003****	**0.424**
	**GFR** [ml/min]	822	96.68	667	97.93	155	91.32	**0.017****	**0.438**
**Male Patients only**	**Erythrocytes** [/pl]	588	4.76	499	4.79	89	4.63	0.235	0.461
	**GFR** [ml/min]	582	100.86	493	101.36	89	98.12	0.489	0.477
**Female Patients only**	**Erythrocytes** [/pl]	241	4.40	175	4.43	66	4.35	0.071	**0.425**
	**GFR** [ml/min]	240	86.54	174	88.22	66	82.13	0.117	**0.435**

Correlations were assessed using the Mann-Whitney-U-Test**
*** positive value for the state variable = high grade toxicity

Abbreviations: No./n, number; GFR, glomerular filtration rate
Bold printed: significant **P<0.05** or **AUC > 0.54, < 0.46**

Supplementary Figure Legend

Figure S1.

(A) Internal cross validation performed with “modelvalid” function in R for Final Model A,

(B) Optimism adjusted AUC calculated with “aucadj” function in R for Final Model A.

(C) Internal cross validation performed with “modelvalid” function in R for Final Model B,

(D) Optimism adjusted AUC calculated with “aucadj” function in R for Final Model B.

(E) Internal cross validation performed with “modelvalid” function in R for Final Model C,

(F) Optimism adjusted AUC calculated with “aucadj” function in R for Final Model C.

(G) Internal cross validation performed with “modelvalid” function in R for Final Model D,

(H) Optimism adjusted AUC calculated with “aucadj” function in R for Final Model D.

(I) Internal cross validation performed with “modelvalid” function in R for Final Model E,

(J) Optimism adjusted AUC calculated with “aucadj” function in R for Final Model E.

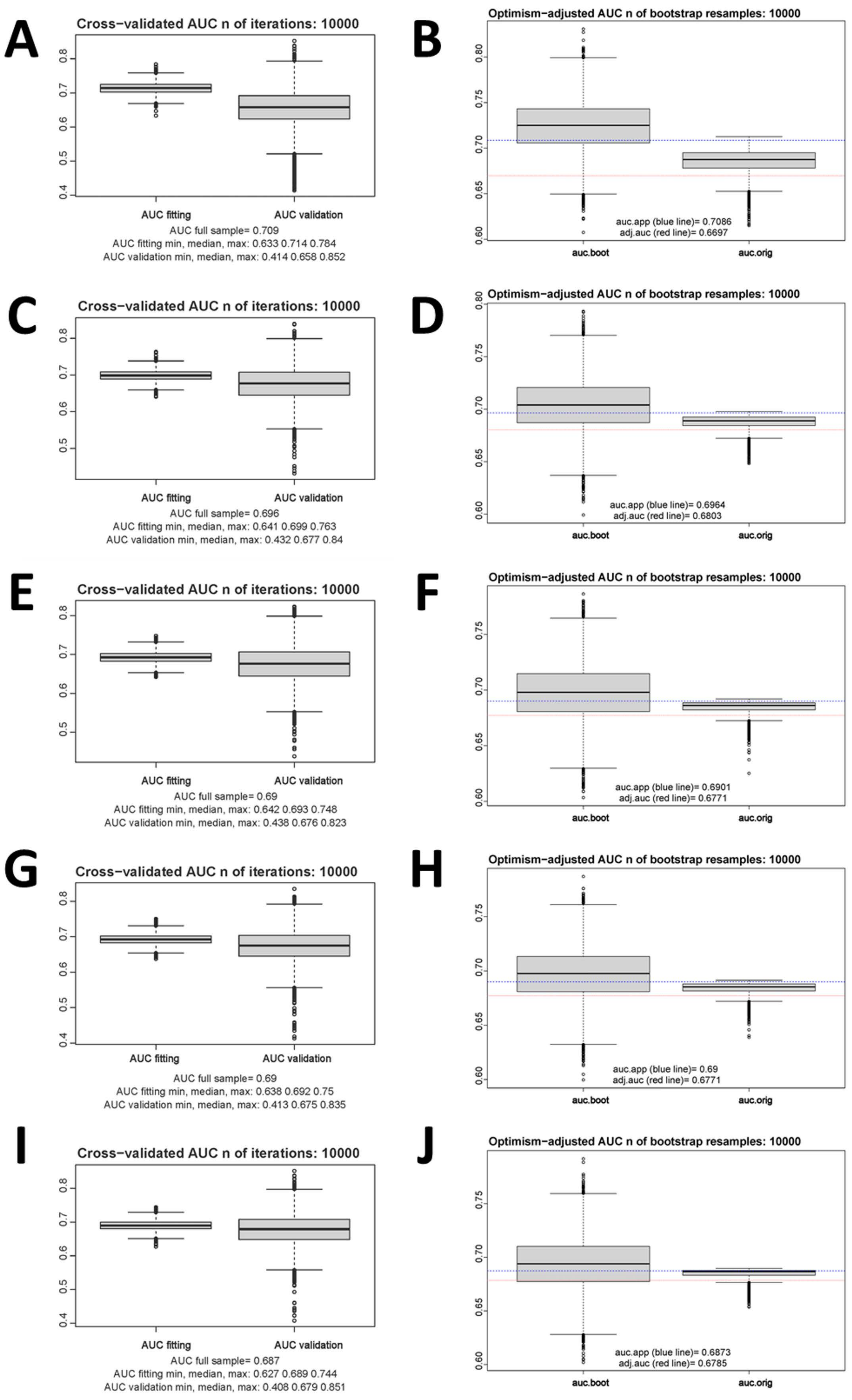
A
Cross−validated AUC n of iterations: 10000
AUC fitting
AUC validation
AUC full sample= 0.709
AUC fitting min, median, max: 0.633 0.714 0.784
AUC validation min, median, max: 0.414 0.658 0.852
B
Optimism−adjusted AUC n of bootstrap resamples: 10000
auc.app (blue line)= 0.7086
adj.auc (red line)= 0.6697
auc.boot
auc.orig
C
Cross−validated AUC n of iterations: 10000
AUC fitting
AUC validation
AUC full sample= 0.696
AUC fitting min, median, max: 0.641 0.699 0.763
AUC validation min, median, max: 0.432 0.677 0.84
D
Optimism−adjusted AUC n of bootstrap resamples: 10000
auc.app (blue line)= 0.6964
adj.auc (red line)= 0.6803
auc.boot
auc.orig
E
Cross−validated AUC n of iterations: 10000
AUC fitting
AUC validation
AUC full sample= 0.69
AUC fitting min, median, max: 0.642 0.693 0.748
AUC validation min, median, max: 0.438 0.676 0.823
F
Optimism−adjusted AUC n of bootstrap resamples: 10000
auc.app (blue line)= 0.6901
adj.auc (red line)= 0.6771
auc.boot
auc.orig
G
Cross−validated AUC n of iterations: 10000
AUC fitting
AUC validation
AUC full sample= 0.69
AUC fitting min, median, max: 0.638 0.692 0.75
AUC validation min, median, max: 0.413 0.675 0.835
H
Optimism−adjusted AUC n of bootstrap resamples: 10000
auc.app (blue line)= 0.69
adj.auc (red line)= 0.6771
auc.boot
auc.orig
I
Cross−validated AUC n of iterations: 10000
AUC fitting
AUC validation
AUC full sample= 0.687
AUC fitting min, median, max: 0.627 0.689 0.744
AUC validation min, median, max: 0.408 0.679 0.851
J
Optimism−adjusted AUC n of bootstrap resamples: 10000
auc.app (blue line)= 0.6873
adj.auc (red line)= 0.6785
auc.boot
auc.orig
